# Improved spatio-temporal measurements of visually evoked fields using optically-pumped magnetometers

**DOI:** 10.1038/s41598-021-01854-7

**Published:** 2021-11-17

**Authors:** Aikaterini Gialopsou, Christopher Abel, T. M. James, Thomas Coussens, Mark G. Bason, Reuben Puddy, Francesco Di Lorenzo, Katharina Rolfs, Jens Voigt, Tilmann Sander, Mara Cercignani, Peter Krüger

**Affiliations:** 1 Department of Physics and Astronomy, University of Sussex, Falmer, Brighton, BN1 9HQ UK; 2 Clinical Imaging Sciences Centre, University of Sussex, Falmer, Brighton, BN1 9PH UK; 3 Physikalisch Technische Bundesanstalt, 10587 Berlin, Germany; 4 Cardiff University Brain Research Imaging Centre (CUBRIC), Cardiff University, Cardiff, CF24 4HQ UK

**Keywords:** Neuroscience, Visual system, Quantum physics

## Abstract

Recent developments in performance and practicality of optically-pumped magnetometers (OPMs) have enabled new capabilities in non-invasive brain function mapping through magnetoencephalography. In particular, the lack of cryogenic operating conditions allows for more flexible placement of sensor heads closer to the brain, leading to improved spatial resolution and source localisation capabilities. Through recording visually evoked brain fields (VEFs), we demonstrate that the closer sensor proximity can be exploited to improve temporal resolution. We use OPMs, and superconducting quantum interference devices (SQUIDs) for reference, to measure brain responses to flash and pattern reversal stimuli. We find highly reproducible signals with consistency across multiple participants, stimulus paradigms and sensor modalities. The temporal resolution advantage of OPMs is manifest in a twofold improvement, compared to SQUIDs. The capability for improved spatio-temporal signal tracing is illustrated by simultaneous vector recordings of VEFs in the primary and associative visual cortex, where a time lag on the order of 10–20 ms is consistently found. This paves the way for further spatio-temporal studies of neurophysiological signal tracking in visual stimulus processing, and other brain responses, with potentially far-reaching consequences for time-critical mapping of functionality in healthy and pathological brains.

## Introduction

Over the last century, outstanding advances in medical physics have led to the development of non-invasive functional neuroimaging techniques^1–3^. This has provided significant insights into brain function and connectivity. Important improvements in modern neuroimaging techniques have allowed neural patterns associated with specific stimulations to be investigated^4^, providing information about the signal’s spatial and temporal characteristics^5^. Previous studies have shown that a spatio-temporal analysis of brain signals is not only essential to understand the basic mechanisms of brain circuits, but would also provide reliable biomarkers for differentiating physiological and pathological brain activity in neurodegenerative diseases^6,7^. There is even a potential for predicting clinical progression or treatment responses^8^. The realisation of the full scope of temporal and spatial localisation of brain signals, however, is hampered by the intrinsically low spatio-temporal resolution of currently available methods^9,10^.

Functional Magnetic Resonance Imaging is capable of mapping activated brain regions with high spatial resolution, but offers only low temporal resolutions ($\sim 1\hbox { s}$), as the local measured changes in blood flow are not synchronized with neuronal activity^11^. Electroencephalography (EEG) is a real-time neuroimaging method, with limited source localisation capability and spatial resolution ($\sim 10\hbox { mm}$)^12^.

Magnetoencephalography (MEG) is an alternative real-time method with a theoretically possible improved spatial resolution, able to measure postsynaptic potentials of tangential pyramidal cells at the surface of the scalp^12^. Recent research has shown that MEG can be used for the evaluation of abnormal cortical signals in patients with Alzheimer’s disease^13^, Parkinson’s disease^14^, autism spectrum disorder^15^, and in severe cases of post-traumatic stress disorder^16^. However, MEG suffers from low signal-to-noise ratio (SnR), and its use is confined to magnetically-shielded rooms (MSRs). The magnetically shielded environments are used to subdue environmental magnetic noise, often many orders of magnitude higher than neuromagnetic fields (fT to pT range).

Traditionally, MEG relies on an array of superconductive quantum interference devices (SQUIDs) to measure the brain’s magnetic fields^17^.

With the sensor array being fixed inside a required cryogenic dewar, the locations of the individual sensors must be arranged to fit a vast majority of head sizes and shapes^18^. The fixed positions result in different radial offsets from a subject’s head. Coupled with tiny head movements from a subject during a measurement, the offsets and fixed positions have a major impact on the potential cortical activity detection^19^. In particular, the theoretically achievable precision of signal source localisation is lost. This makes SQUID-MEG impractical in many cases, in particular in the clinical context.

Extremely sensitive spin-exchange relaxation-free (SERF) optically-pumped magnetometers (OPMs), developed at the turn of the millennium^20^, can help to overcome the SQUID-MEG limited spatial resolution^21^. With the OPMs able to be fixed to a subject’s head^22^, a smaller offset distance than SQUIDS, and the ability for simultaneous dual axis measurements, OPM-MEG has several advantages over SQUID-MEG, including its suitability for applications within pediatric and clinical populations.

The aim of this study was to demonstrate the improved ability of OPM-MEG by recording spatio-temporal characteristics of neurophysiological signals, and comparing them to conventional SQUID-MEG. As a prototypical test case we have chosen visual cortex responses to established standard visual stimulations, with the measured responses evaluated in a well-characterised context. We find that OPM-MEG is superior to SQUID-MEG in brain signal tracking in space and time,making a suitable method to provide new information about propagating signals, source localisation, neural speed, and brain circuits far beyond the processing of visual stimuli.

## Theory considerations

Precisely determining the time onset of magnetic sources is equally important as localising their position when tracking neural activity throughout the brain. The extent to which it is possible to distinguish both location and timing of neurophysiological events is referred to as spatio-temporal resolution. Here we argue that OPM-MEG can be advantageous in both necessary aspects, i.e. in terms of spatial and temporal resolution.

### Spatial resolution

A general definition for spatial resolution in MEG is the minimum separation required for two magnetic sources to be resolved. As the magnetic field amplitude decays according to a power law with the distance from a field source, improved signal detection is achieved when sensors are moved closer to the brain. The consequences of the field decay law are that closer positioning of a sensor system provides improved signal-to-noise, better spatial resolution and more precise source localisation, as shown formally for a generic situation^23^ and confirmed through realistic brain anatomy simulations^24,25^. Quantifying this improvement is only possible in specific situations where source distances and their magnetic field characteristics (multipole expansion) are known. In general, when applying the Rayleigh criterion for resolution, the maximum distance at which two sources can be resolved is comparable to the distance between the two sources^23^. As OPMs can be placed closer to the head than SQUID systems, the OPMs are able to achieve a higher spatial resolution.

### Temporal resolution

We define temporal resolution of an OPM-MEG system as the minimum time interval between two neurophysiological events in the brain to be detected as distinct from each other. Note that this is different from a sensor-level definition that would for example be based on the bandwidth of the device or the sample frequency of the data acquisition system (although these measures can affect the temporal resolution of the MEG system). A given detected event is associated with a magnetic field pulse shape whose measured temporal width, amplitude and signal uncertainty will determine the temporal resolution by the above definition.

The uncertainty of a typical response to a given brain stimulation in MEG is determined through measurements of a series of pulses, commonly averaged over many trials. This averaging over uncorrelated measurements enables association of a statistical standard error with the mean signal determined at each point in time. A practical quantitative definition of temporal resolution is then the time that passes after a characteristic feature (typically a peak) before the signal significantly differs from its value at that characteristic feature.

As a simple and typical example, consider a pulse with a Gaussian shape *g*(*t*):

1 $$\begin{aligned} g(t)=Ae^{\frac{(t-t_0)^2}{2\sigma ^2}}, \end{aligned}$$

where *A* is the amplitude of the pulse, $t_0$ is the time when the pulse maximum occurs, $2\sigma$ is the width of the pulse. The uncertainty of the signal is the standard error $\varepsilon$, assumed to be time-independent in this example. The temporal resolution $t_\mathrm {res}$ for this signal shape is then the time interval between the peak time $t_0$ and the time (after or before) $t_0$ by which the signal is significantly, i.e. by an amount $\varepsilon$, smaller than the peak amplitude *A*, so that

2 $$\begin{aligned} g(t_0)=g(t_0\pm t_\mathrm {res})+\varepsilon \end{aligned}$$

We can solve for $t_\mathrm {res}$ finding,

3 $$\begin{aligned} t_\mathrm {res}=\sigma \sqrt{-2\ln {\left( 1-\frac{\varepsilon }{A}\right) }}. \end{aligned}$$

Note this only holds with $\varepsilon /A<1$. The inverse ratio can be interpreted as the signal to noise ratio $\mathrm {SnR}=A/\varepsilon$ (with $\mathrm {SnR}>1$). In first order Taylor expansion (in the logarithmic term) Eq. (3) simplifies to

4 $$\begin{aligned} t_\mathrm {res}=\sigma \sqrt{\frac{2\varepsilon }{A}}. \end{aligned}$$

This scaling of $t_\mathrm {res}\propto w/\sqrt{\mathrm {SnR}}$ holds also for more general (not necessarily Gaussian) pulse shapes with width *w*. For MEG signals comprising of multiple non-Gaussian pulses it is not always possible to achieve accurate (Gaussian or other functional curve) fits. Within the work presented here, we hence define the width *w* as the time between the two local minima adjacent to a pulse’s maximum signal value, and the amplitude as the difference between the maximum and the mean of those two minima (see inset of Fig. 2).

A consequence of the above scaling is that a measurement method that increases the measured amplitude of a pulse whilst retaining a similar width and standard error, will improve the temporal resolution. OPMs have recently been shown to approach noise floors similar to that of SQUIDs^26,27^, therefore similar values of $\varepsilon$ can be assumed for both technologies. The figure of merit for improving time resolution of MEG then becomes $\eta =\sqrt{A}/w$ with higher values of $\eta$ corresponding to better time resolution, i.e. shorter $t_\mathrm {res}$. OPMs due to their closer proximity to the brain sources measure larger amplitude signals and are hence expected to achieve better values of $\eta$ than SQUIDs.

### Vectoral measurements

In conventional MEG only one component of the vectorial magnetic field is measured. Most commercial setups for SQUID-MEG only measure magnetic field gradients radial to the brain. At the typical stand-off distances of several centimetres, the orthogonal components tend to be weak, so that the radial field (gradient) component approximates the total field (gradient). With closer sensor proximity to the brain, OPMs are able to measure multiple field (gradient) components to extract additional spatial information^28^. A vector measurement taken at short distances does not suffer from the zone of a vanishing field component in the immediate vicinity of a current dipole, and is sensitive to volume currents in the brain.

Measuring both radial and tangential field components also helps to improve signal *temporal* resolution. This is a consequence of the ability to characterise the field as a vector. At the sensor, the magnetic field has a direction and magnitude. A radial sensor measures the magnetic field projected onto the radial direction. By measuring in only the radial direction it is not possible to differentiate between a rotation or a change in magnitude of the magnetic field vector. Worse still, if the magnetic field vector simultaneously changes in both direction and magnitude, then the time at which the magnetic field reaches peak magnitude can be obscured. By measuring a second component of the magnetic field we can begin to differentiate between a change in the magnitude of the magnetic field, and a change in magnetic field direction. Sensors near the head are in a source-free region ($J=0$) , therefore using Ampère’s Law $\nabla \times B = \mu _{0}J=0$ the third magnetic field component can be calculated from the other two magnetic field components assuming the gradient of the magnetic field can be calculated. For a system with a low sensor count, all three magnetic field components need to be measured to achieve full characterisation of the magnetic field.

## Materials and methods

### Participants and MRI

**Figure 1:**
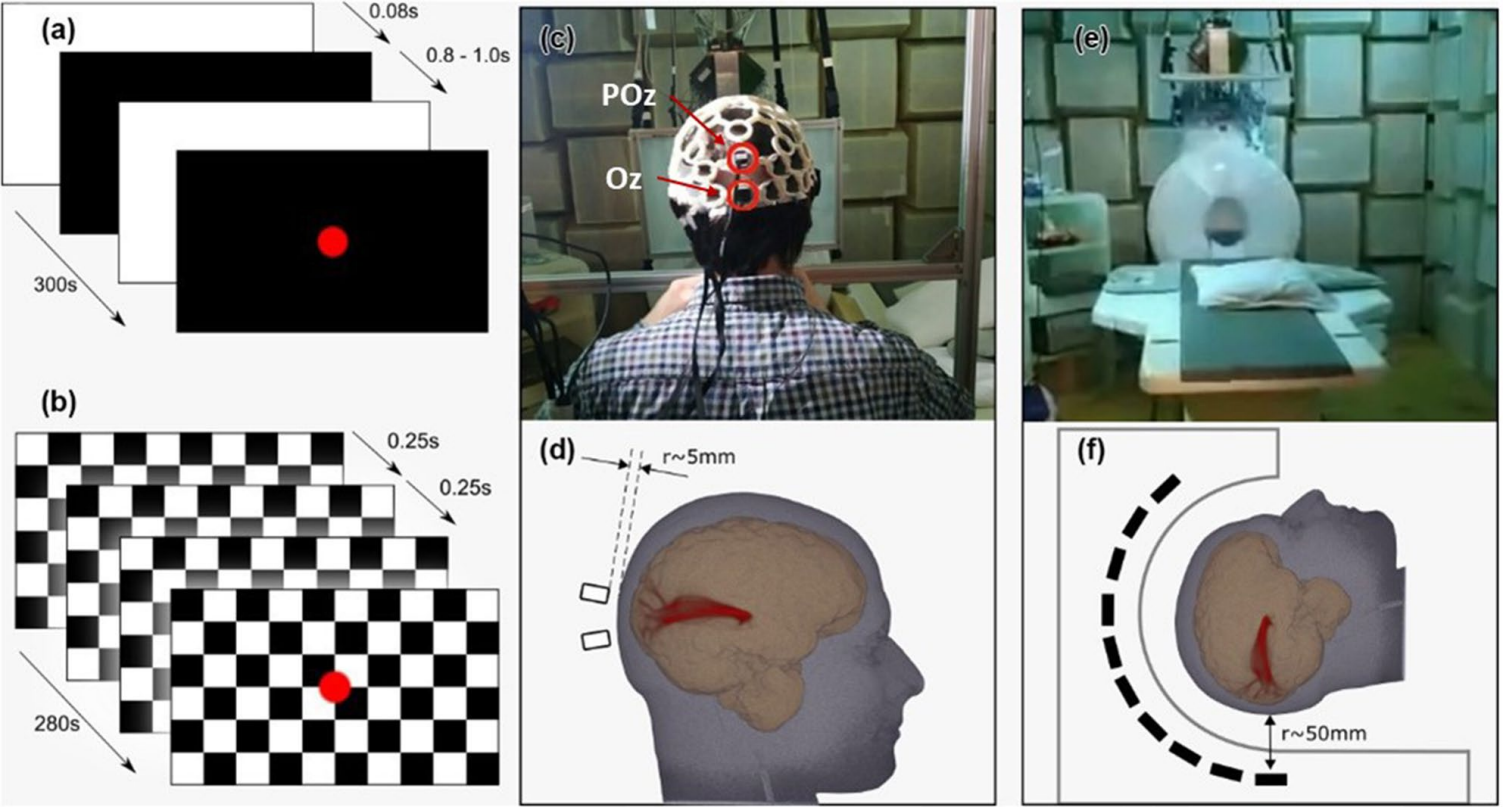
(**a**) Flash and (**b**) pattern reversal stimulation protocols. (**c**) A participant in position with the 3D printed helmet containing the OPM devices. Red highlighted cells show the sensor locations used for the study, the Oz and POz. The sensors were placed over the primary visual cortex and the associative visual cortex, respectively. (**d**) 3D rendering of the MRI scan of Participant 1 showing approximate locations of OPM sensors 1 & 2, and scalp-sensor separation of around 5 mm. The reconstructed optic radiation are also shown in red. (**e**) The Yokogawa SQUID-MEG system. (**f**) Schematic of the SQUID-MEG system showing a sensor to scalp separation of approximately 50 mm.

Visual evoked fields were studied in 3 healthy participants (2 men aged 26 and 30, 1 woman aged 47 years) with normal or corrected-to-normal vision. The 3 participants received a 3 T MRI scan (Siemens Magnetom Prisma, Siemens Healthineers, Erlangen, Germany) at the University of Sussex, including a high-resolution T1-weighted anatomical scan. For one participant a diffusion-weighted scan was acquired for reconstructing the optic radiations, with two diffusion-weighting shells (b values = 1000 and 3000 s/$\hbox {mm}^{2}$). For each b value, diffusion gradients were applied along 60 non-collinear directions. Six images with no diffusion weighting (b = 0) were also collected. Image processing was performed using tools from the FMRIB’s Diffusion Toolbox 5.0. First, data were corrected for involuntary motion and eddy currents using affine registration. BEDPOSTx was run with default settings to fit a crossing fibers model^29^, and finally, XTRACT was used to automatically reconstruct the left and right optic radiations in native space by probabilistic tractography^30^. The results are shown in Fig. 1d,f.

### Experimental design

The study was conducted in accordance with the Declaration of Helsinki Ethical Principles, and was approved by the Brighton and Sussex Medical School Research Governance and Ethics Committee (ER/BSMS3100/1); all participants gave written informed consent to take part, after explanation of the procedure and purpose of the experiment. All MEG measurements, OPM-MEG and SQUID-MEG, were taken in the Ak3b MSR (Vacuumschmelze, Hanau, Germany) at Physikalisch- Technische Bundesanstalt (PTB), Berlin. The MSR is equipped with an external triaxial active shielding coil system controlled by fluxgates. Inside the MSR field fluctuations are sufficiently weak to allow OPM operation^31,32^.

Two standard full-field visual stimulation protocols were employed during the MEG recording, a flash stimulus (FS), and a pattern reversal stimulus (PR). The parameters used were based on standard guidelines for clinically evoked potentials^33^. These paradigms are widely used to evaluate early visual processing, and to detect abnormalities in the visual pathways. The flash stimulus, shown in Fig. 1a, consists of short white flashes of length 0.08 s (5 frames). To avoid participants from preempting the stimulus, each white flash was followed by a dark period with the length varying pseudo-randomly between 0.92 s and 1.00 s (55 to 60 frames). The total duration of a single FS measurement run was 300 s.

The pattern-reversal stimulus, Fig. 1b, consisted of a black and white checkerboard (10 squares wide, 8 high) with the colours inverting at 0.5 s (30 frame) intervals. Each run had a duration of 280 s. For both FS and PR, a red dot was continuously projected onto the centre of the screen to act as a focal point for the participant. Before each measurement run, whilst in position for the trial, the participants were exposed to a three-minute dark adaptation period. Measurements of the empty MSR were obtained in order to evaluate environmental noise levels. During the noise measurements, the OPMs were located in the same position and orientation as they would with a participant wearing the sensors. During measurements, participants sat upright with the sensors mounted in a 3D-printed helmet (Fig. 1c). A chin rest was used to help stabilise each subject’s head, reducing movement when looking forward at a 50 cm $\times$ 34 cm vertically orientated screen. The stimuli were projected on to the screen via a mirror system using a 60 Hz LCD projector, positioned outside of the MSR. The SQUID-MEG system (Fig. 1e) accommodated participants in a horizontal position, with the same screen positioned horizontally above the subject. The screen to eye distance was 53 cm for the OPM-MEG setup and 45 cm for the SQUID-MEG system.

## MEG systems overview

### OPM-MEG

The OPM-MEG system consisted of two second-generation QuSpin zero-field magnetometers (QuSpin Inc., Louisville, CO, USA), with a specified typical sensitivity $<15\hbox { fT}/\hbox {Hz}^{1/2}$ and magnetic field measurement bandwidth of 135 Hz in a 12.4 mm $\times$ 16.6 mm $\times$ 24.4 mm sensor head. The OPMs were mounted in a 3D printed helmet (open-source design; OpenBCI Mark IV helmet) (https://github.com/OpenBCI/Ultracortex/tree/master/Mark_IV/MarkIV-FINAL) and positioned over the visual cortex at the Oz and POz positions, according to the standard 10–10 system^34^. These locations were chosen in accordance with each subject’s MRI scan.

The Oz and POz positions correspond to the primary visual cortex (V1) and the associative visual cortex (V2), respectively. Studies have shown the feed-forward and feedback interaction between the V1 and V2 areas in response to visual stimulation^35^. More specifically, there is an early activation at V1, known as the P1 or C1 component, which is then suppressed as the signal propagates to V2, after which a reflected wave is initiated and propagates back to V1^36^.

The design of the helmet and sensor head fixes the scalp to sensor distance to $\sim 5\hbox { mm}$. Python-based software was developed for the design and presentation of stimuli. The software was directly connected and synchronized with a main OPM-MEG data acquisition system (DAQ). The OPM-MEG system’s analogue outputs were recorded at 1 kHz via a Labjack T7 pro (Labjack Corporation, Co, USA). All DAQ electronics, except the OPM sensor-heads, were located outside the MSR and directly connected to the Labjack and control computer.

### SQUID-MEG

The SQUID-MEG system MEGvision (Yokogawa Electric Corporation, Japan) comprises of 125 axial gradiometers and 3 reference magnetometers. For the stimuli presentation the same software was used to prevent any bias in the stimulation delivery. Data from all sensors were recorded at a 2 kHz sampling frequency, and the sensors located closest to the OPM positions were used for analysis. The fixed positions of the sensors result in a $\sim 50\hbox { mm}$ standoff from the subject’s scalp. The SQUID-MEG used MEG Laboratory 2.004C (Eagle Technology Corporation) data acquisition software.

### Data analysis

The DAQ systems were synchronised with the presentation software. Both OPM and SQUID data analyses were performed using the FieldTrip toolbox^37^ and MATLAB. In order to isolate the frequencies of interest with relevance in visually evoked fields, all data were filtered with a bandpass filter between 5 and 60 Hz. A further bandstop filter was applied between 49 and 51 Hz to suppress 50 Hz line-noise. The epoched trials for FS were $-45\hbox { ms}$ to 350 ms, and 0–250 ms for PR. Any trials with interrupted recordings were removed from the analysis. All the time-locked averaged responses contain more than 380 trials for the FS stimulation, and more than 280 for PR.

In the following sections the evoked fields are shown as the mean across all individual trials for a single run. The uncertainties on the signal amplitudes are calculated as the standard error at each time point (with a 1 ms time spacing for OPM-MEG and 0.5 ms for SQUID-MEG).

The resulting uncertainty band is then used to determine temporal uncertainties of signal features such as amplitude peaks. The time error is set as the width of the uncertainty band at the amplitude feature, as outlined in “Temporal resolution” section.

In order to compare the spatio-temporal response of OPM-MEG to SQUID-MEG we initially study the temporal resolution of the two systems by measuring $\eta$ in characteristic evoked magnetic field peaks as defined at the the theoretical considerations “Temporal resolution” section. The $\eta$ uncertainty results as error propagated from time and signal uncertainties, determined in the above described manner.

In a second step, the evoked potentials as measured at the two sensor locations are then compared. For the OPM system, the simultaneously obtained individual field component (radial $B_z$ and axial $B_y$) data are further compared to the resulting planar projection $B_{yz}$, with $|B_{yz}|=\sqrt{B_{y}^2+B_{z}^2}$. This reduces timing artefacts that can occur in data restricted to a single component.

VEFs are characterized by three time components occurring at different times: the early component (P1), the main component (P2 for flash stimuli and P100 for pattern reversal stimuli), and the late component (P3), where we established the onset range for the main and late components based on previous studies^10,38–42^. The flash and pattern reversal stimulus responses consist of an early component with peak onset between 35 and 60 ms, a main component (P2) between 83 and 152 ms, and a late component (P3) between 160 and 230 ms.

Each participant had at least four FS runs and three PR runs with the OPM-MEG system, and a single run for each stimuli with the SQUID-MEG.

The averaged responses were determined using the same method as detailed above.

## Results

In total, 30 OPM-MEG runs were undertaken, with 12 FS, 9 PR, and 9 background runs. In addition, a single FS and PR run was conducted with SQUID-MEG for each participant. The VEFs from all participants and all modalities were consistent with patterns known from the literature.

**Figure 2:**
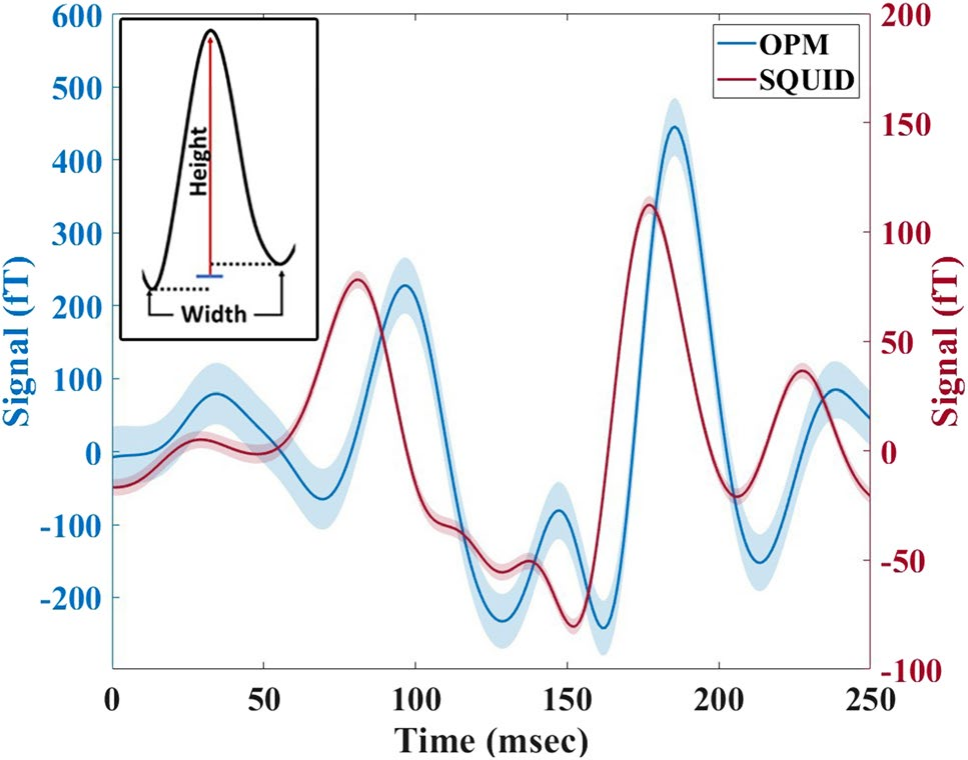
Averaged evoked field recorded by OPM-MEG and SQUID-MEG for Participant 1 for flash stimulation over a single measurement run (300 trials). VEF measured at Oz using OPM sensor (blue line) and the corresponding SQUID sensor (red line).The shaded area shows the standard error. Inset: The signal height or A (red line) is the amplitude difference between the peak maximum and the mean of the two local minima (blue line). The width or w is the time difference between the two local minima (dashed lines). The $\eta$ is the ratio $\sqrt{A}/w$ of two values.

We consider the $\eta$ of the two systems and the higher SnR of OPM-MEG observed in other studies^22,24^ compared to SQUID-MEG. Figure 2 shows measurements from a single OPM sensor and the corresponding SQUID sensor for FS. The OPM-MEG recorded signals with up to 4 times higher amplitude than the SQUID-MEG, with the OPM and SQUID sensors recording a maximum amplitude of 480(46) fT and 126(4) fT, respectively. Along with the increase in amplitude over the SQUIDs, we see the same activation patterns in both methods, further verifying the OPM’s recorded traces. The OPM $\eta$ was found to be $0.5(6)\,\sqrt{\hbox {fT}/\hbox {ms}}$, compared to the SQUID $\eta$ of $0.25(2)\,\sqrt{\hbox {fT}/\hbox {ms}}$. In spite of having a non-optimal noise floor at our OPM DAQ system, in our case the higher $\eta$ still indicates a higher temporal resolution of the OPM-MEG neuroimaging system. The OPM VEF shows more pronounced peaks, resulting in sharply defined maxima and minima, resulting in lower temporal uncertainties compared to SQUID-MEG measurements.

**Figure 3:**
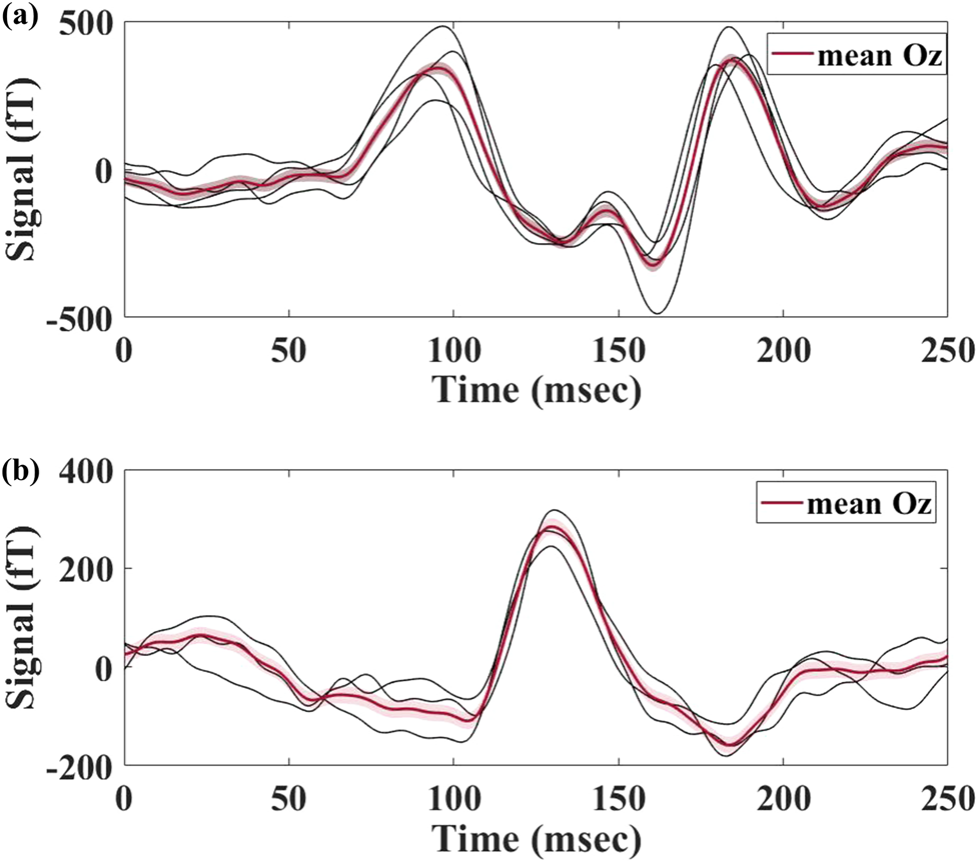
OPM VEF for (**a**) four FS runs and the associated mean. (**b**) Three PR runs along with the mean for Participant 1. The individual runs (black) for both FS and PR show the same activation pattern as the associated mean (red). The shaded area displays the standard error of the mean.

In Fig. 3 we plot all participant 1 PR and FS runs recorded by OPM-MEG at Oz, along with their average. The individual runs illustrate the reproducibility of the activation patterns during both stimulations, with the main (P2 or P100) and late components (P3) having similar time onsets across all runs. For FS, the main component (P2) has an onset time between 135 ms and 100 ms and the late component (P3) between 180 ms and 190 ms. For PR, the main component P100 occurs between 128 ms and 133 ms, while the late component (P3) occurs between 210 ms and 214 ms.

Visually evoked responses recorded for FS and PR had similar activation patterns and onset delays across all runs for each participant (Table 1). Participant 1 displays a similar activation pattern for all the FS and PR runs. Participant 2 had well defined and reproducible FS responses, while the PR responses showed slightly more variation. Participant 3 displayed similar activation patterns for both FS and PR. Anatomical differences of the cortical surfaces of each participant could be the origin of the small variability of the evoked responses between the participants and the inverse correlation (see Table 2) between participant 2, participant 3 and participant 1. All participants had VEF with similar onset patterns and differences for the signal times at the POz and Oz sensor locations.

**Table 1:** Pearson correlation coefficient across 4 FS runs and 3 PR runs for each participant.

Stimulation	Flash stimulus	Pattern reversal
Participant 1	0.83 (4)	0.85 (2)
Participant 2	0.70 (7)	0.24 (8)
Participant 3	0.56 (3)	0.54 (6)

The bracketed values are the standard error.

In order to quantify the within-participant reproducibility of VEFs qualitatively observed in Fig. 3, we calculate the Pearson correlation coefficients between runs per participant  1. The correlation coefficients for the flash stimulus and pattern reversal were calculated to be 0.83 and 0.85 for participant 1, respectively, and 0.70 and 0.24 for participant 2, and 0.65 and 0.54 for participant 3.

In addition to this, we then calculate the correlation coefficient between subjects for the same stimuli (Table 2). Moderate between-subject correlation coefficients were found for participants 2 and 3, while Participant 1 showed anti-correlated signal at both sensors. The different cortical folding of each participant could explain the anti-correlation measured between the VEFs. Previous studies have shown an asymmetry in extracranial magnetic field measurements due to variabilities in cortical folding^43,44^.

**Table 2:** Pearson correlation coefficient between participants for Flash and Pattern reversal stimulation recorded at the Oz sensor.

Participants/stimuli	Flash stimulation	Pattern reversal
Participant 1–Participant 2	− 0.53 (7)	− 0.45(9)
Participant 1–Participant 3	− 0.54 (7)	0.49 (9)
Participant 2–Participant 3	0.38 (8)	− 0.35 (11)

The bracketed values give the 95% confidence interval.

**Figure 4:**
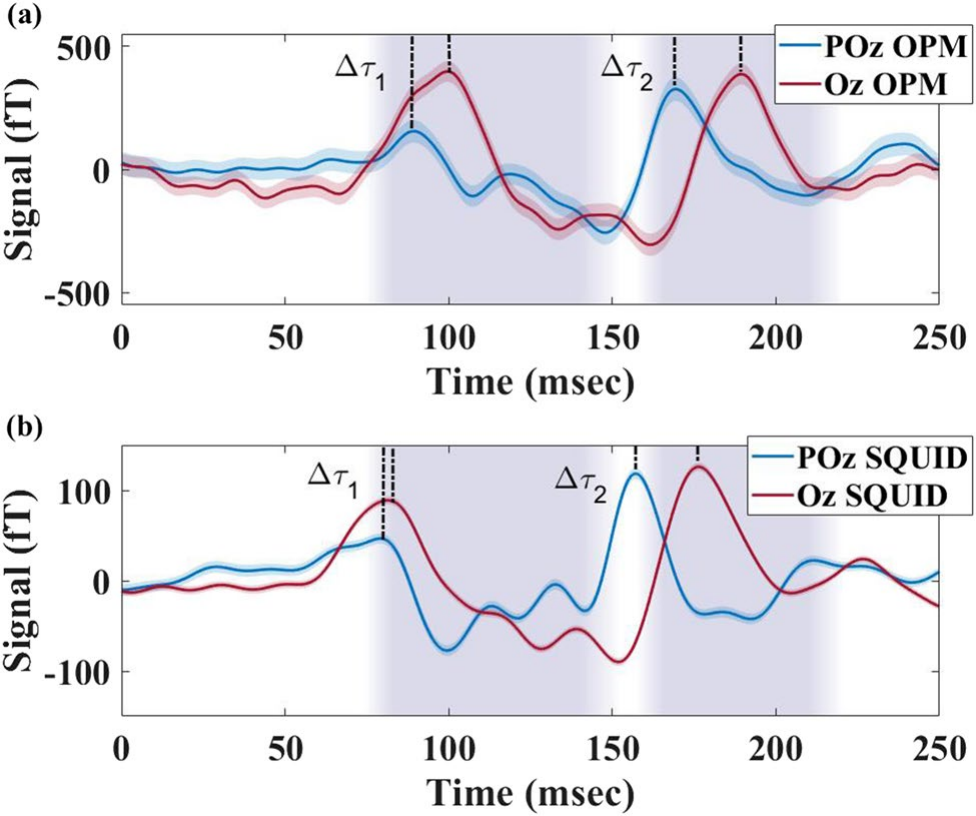
Visually evoked response during flash stimulation recorded by: (**a**) OPM-MEG and (**b**) SQUID-MEG for Participant 1. The coloured areas indicate the limits where the peak onset for Oz is expected for each stimulus^10,38–42^. The selected peaks for Oz (red) and POz (blue) sensors are marked with dashed lines for both components P2 and P3.

Figure 4 displays a single run recorded by OPM-MEG (a) and SQUID-MEG (b) systems during FS. Although OPM-MEG shows the initial activation (P1) at the primary visual cortex, there is a significant time difference between the arrival of the signal at POz and Oz for both the main and late components, with an earlier activation at POz. The vertical purple bands represent the time range of P2 and P3 components found in previous studies for Oz EEG sensors^9,10,38–42^. The dominant peaks that fall within these boundaries are shown by bold dashed lines, representing the peak times of the main (P2) and late (P3) components. $\Delta \tau _1$ and $\Delta \tau _2$, defined as the delay between signals arriving at POz and Oz for the main and late components, were measured as 10(7) ms and 20(4) ms, respectively.

The earlier activation of POz compared to Oz for Participant 1 was observed in all four runs, with $\overline{\Delta \tau _1}=$ 8(1) ms and $\overline{\Delta \tau _2}=$ 18(1) ms (see Fig. 6).

The reproducibility of the time delay, and the small variations in $\Delta \tau _1$ and $\Delta \tau _2$ over multiple measurement runs points to a neurophysiological origin of the delay, such as a timing difference of signals arriving at different locations within the visual cortex. While the absolute timings differ from those determined with the OPMs, similar time delays are also found for the SQUID-MEG measurement, where we find $\Delta \tau _1=$ 2(5) ms and $\Delta \tau _2=$ 18(3) ms (Fig. 4b). The difference in absolute timings between OPMs and SQUIDs is not unexpected, as the precise positioning of the sensors differ both laterally and by distance from the scalp. Furthermore, the orientation of the sensitive axis for the SQUIDs is not fully aligned with the radial component measured by the OPMs. Although we recorded a higher SnR for SQUID, over OPM measurements, the timing uncertainties for $\Delta \tau _{1,2}$ are similar in both modalities due the improved $\eta$ achieved with OPMs.

**Figure 5:**
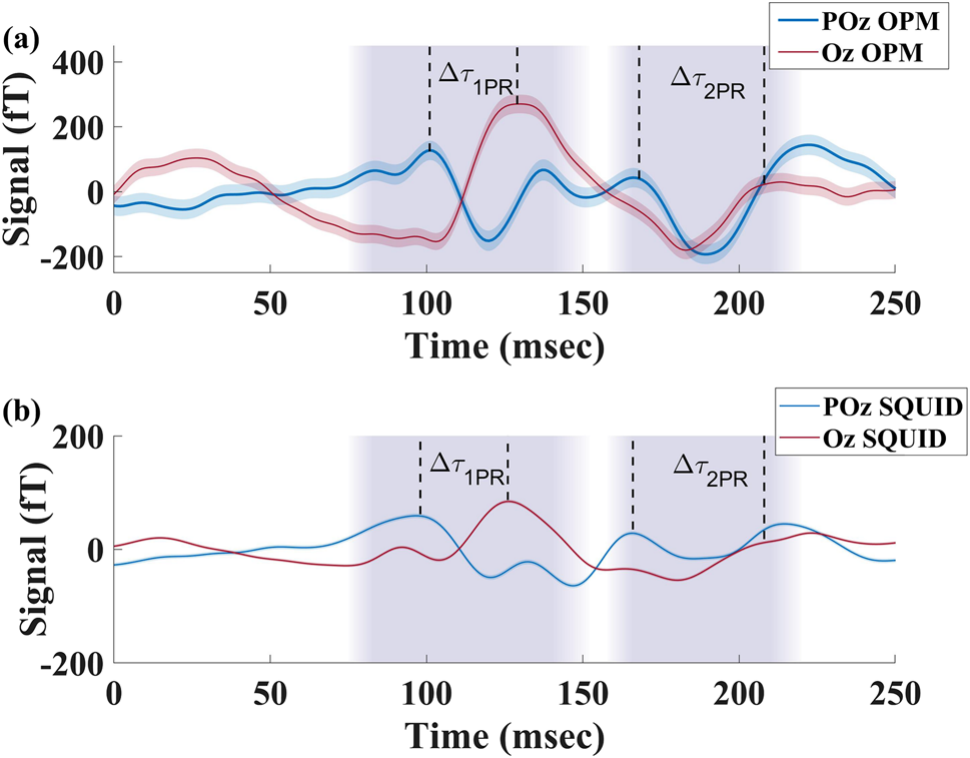
Visually evoked response during pattern reversal stimulation recorded by: (**a**) OPM-MEG and (**b**) SQUID-MEG for Participant 1. The coloured areas indicate the limits where the peak onset for Oz is expected for each stimulus^10,38–42^. The selected peaks for Oz (red) and POz (blue) sensors are marked with dashed lines for both components P2 and P3. The selected peaks for Oz (red) and POz (blue) sensors are marked with dashed lines for both components P2 and P3.

**Figure 6:**
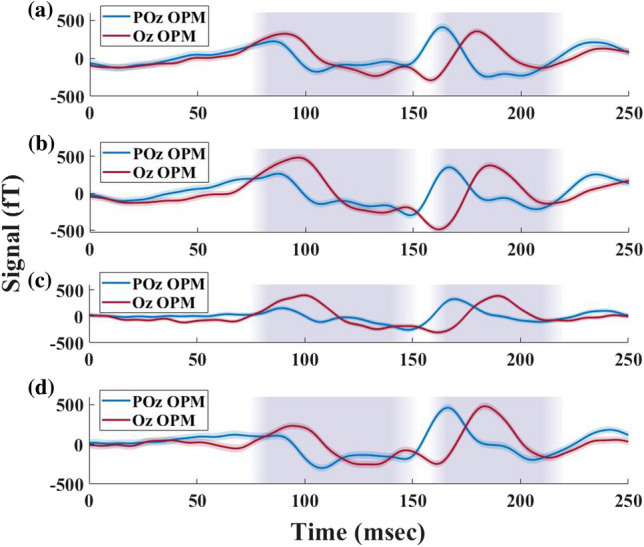
OPM VEF between the POz and Oz sensor for four flash stimulation runs for Participant 1. All four runs show similar peak onset and amplitude. The time lag between the POz (blue) and the Oz (red) OPM sensors is consistent for the two components across the runs.

We observe the same activation delay for the pattern reversal stimulation for participant 1 (Fig. 5). The time delays were measured as $\Delta \tau _{1PR}=$28(16) ms and $\Delta \tau _{2PR}=$45(8) ms. Although $\Delta \tau _1$ and $\Delta \tau _2$ are observed in all the evoked responses during both FS and PR, the time delays during PR stimulation are significantly longer than the FS. Similar activation pattern and delays are observed across all the PR runs. SQUID-MEG recorded similar time delays for the PR stimulus, with the $\Delta \tau _{1PR}$ as 30(6) and $\Delta \tau _{2PR}$ as 57(5). The observed activation patterns were shown to be reproducible across all runs (Fig. 6) and stimuli (Fig. 4), with activation of POz before Oz detected in all participants.

**Figure 7:**
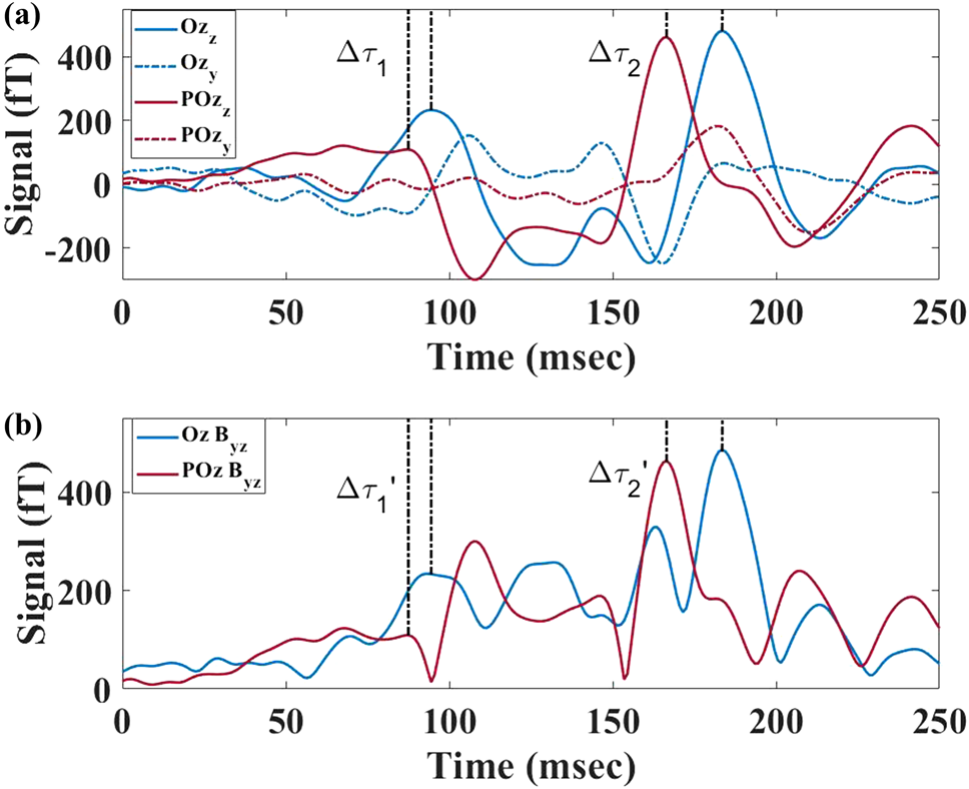
FS VEF recorded at Oz (blue) and POz (red) using OPM-MEG. (**a**) The averaged Oz and POz response along the *z* (bold) and *y* (dashed) directions. (**b**) The Oz and POz magnitude projected into the y-z plane. The bold black lines indicate the earlier activation of POz followed by the Oz.

Compared to SQUIDs, the additional feature of OPM-MEG to simultaneously measure components along two axes, in this case *y* and *z*, can be used to further support the neurophysiological origins of the delay phenomenon, such as those observed here for the signal’s main and late components. Figure 7 shows the two magnetic field components $B_y$ and $B_z$ simultaneously measured by two OPMs, along with each sensor’s magnitude projected in the *yz*-plane $|B_{yz}|$.

In Fig. 7a we show OPM-MEG $B_{y}$ and $B_{z}$ FS responses recorded simultaneously from a single run at POz and Oz. $B_z$ shows a VEF with higher amplitudes and more clearly discernable peak structure than that recorded by $B_y$. In Fig. 7b we show $|B_{yz}|$. The characteristic components of the VEF recorded in the vector components persists, including the timings and relative time delays of the main VEF features (previously negative peaks are now positive as the *yz*-plane projection is displayed as the modulus). Our result of a significant and reproducible time delay between signals arriving at POz and Oz (Figs. 4 and 7) is consistently observed across participants and stimuli.

## Discussion

In this study we used VEFs to assess and demonstrate the ability of MEG based on two types of highly sensitive magnetometers (OPMs and SQUIDs) to detect neurophysiogical brain signals with simultaneously high spatial and temporal resolution. We find that both sensor modalities are suitable to reproduce characteristic brain signatures known from well-established neurophysiological research and clinical practice.

The ability to track local brain responses in space and time can be quantified by determining the time interval over which a signal rises and falls. We find that the OPM $\eta$ has a twofold increase over SQUID measurements, confirming the expectation of the closer proximity of the OPMs to the visual cortex having such an effect.

Importantly, we were able to confirm that the OPM-MEG measurements are robust. Repeating the experiment with two different visual stimuli (flash stimulus and a checker board pattern reversal stimulus) and with three different participants, we observed good reproducibility over multiple repeated runs within each subject and each stimulus. Differences between subjects and type of stimulus are discernable, but the key signal characteristics remain. Individual cortical folding variations could lead to different cancellation of the extracranial magnetic field^43,44^ which reflects the asymmetry of the VEF and the anti-correlation between some of the participants’ responses.

Finally, we illustrate that OPM-MEG is capable of recording neurophysiological signals of a common origin at different locations at different times. By measuring the arrival times of characteristic VEFs at two distinct locations within the visual cortex. The temporal resolution is sufficiently high to determine significant time differences between the primary visual cortex (Oz) and the associative visual cortex (POz). We measured a consistent delayed response at the Oz position relative to the POz position on the order of 10 to 20 ms for the second (P2/P100) and third (P3) components. This observation is again highly reproducible for different runs and is similar across participants and both types of stimuli. It is confirmed by corresponding SQUID measurements. The time delay uncertainties of the OPM data are comparable and even slightly lower than their SQUID counterparts. We attribute this to the strongly enhanced $\eta$ featured by the OPMs.

In order to verify the neuronal nature of the measured time delay, we analysed the recordings along the OPM’s orthogonal *y* and *z* axes. Although we have already demonstrated that our analysis of $B_z$ results in an earlier activation of POz, the inclusion of a second axis, for which we now measure $|B_{yz}|$, follows the same trend. We can indicate that the observed activation pattern is more likely to be from neural activity than an artifact of limited information. The future addition of a third orthogonal axis, to complement our two axis system, will be beneficial in order to fully validate the activation source observed. As we have demonstrated the reproducibility of VEFs in separate runs, sometimes recorded over different days, acquiring three dimensional recordings by rotating the OPM-MEG sensors between runs could be used in future experiments.

Although the VEFs are well defined in humans^41^, the spatio-temporal pattern of the propagating signal is not well characterized. Studies have previously revealed the interaction of the primary visual cortex (V1) with associative visual areas (V2, V3) using an invasive cortical feedback system in animal models^35,36^. The hierarchical order and spatio-temporal processing of the signal in humans remains uncertain. Some studies have claimed P1 originates from the primary visual cortex^10,45^, while others indicate it originates from the extrastriate cortex^38,46^. Additionally, the P2/P100 component appears to originate from the extrastriate cortex without a definite region^10^. The widespread sensor positioning of electrodes or SQUIDs combined with the low spatio-temporal resolution may not be able to record coincident responses from close cortical sources. Here, we introduce the OPM-MEG system as a non-invasive investigational tool, with the potential to further detail and explore the structural and functional connectivity of neighbouring cortical areas, with a higher spatio-temporal resolution than currently available. Our initial experiments are consistent with the findings in animal models^35,36^ being applicable also to the human brain.

The benefits of OPM-MEG could be important both at research and clinical levels: its higher spatio-temporal resolution would allow to better investigate neural networks, shedding light on the relationships between the connectivity of functionally related brain areas, along with their frequency synchronization. Moreover, this advancement could be applied in clinical populations at different stages, such as those with Alzheimer’s disease. In patients with mild cognitive symptoms, topographical biomarkers based on the analyses of the frequency domain might monitor the progression of the disease over years and help to evaluate therapy response. An even higher impact could be achieved especially at a prodromal (or, even better, preclinical) stage, in which these biomarkers could be used as “gatekeepers” for people at risk of developing Alzheimer’s disease^47^.

As this study’s small sample was limited, future research should aim to demonstrate the reproducibility of our results with a larger population. Moreover, it is important to explore the high spatio-temporal resolution of the OPM-MEG system using different stimuli and explore the propagating signals of different brain circuits. Further research is needed to investigate other sensitive pathways in order to better establish the suitability of OPM-MEG for its application in neurophysiological studies. In addition, we have subsequently shown that a factor-six improvement in the DAQ noise floor can be achieved, increasing the SnR of the OPM-MEG for an even higher spatio-temporal resolution.

Based on our observations, OPM-MEG could be a reliable neuroimaging method to identify the activation patterns of close cortical regions in response to specific stimuli. It has the potential to enable reliable neural speed measurements, and spatio-temporal tracking, of propagating signals, including more detailed investigations of the visual pathway.

## References

[1] Berger H (1929). Über das Elektrenkephalogramm des Menschen. Archiv für Psychiatrie und Nervenkrankheiten.

[2] Cohen D (1972). Magnetoencephalography: Detection of the brain‘s electrical activity with a superconducting magnetometer. Science.

[3] Ogawa S, Lee T-M, Nayak AS, Glynn P (1990). Oxygenation-sensitive contrast in magnetic resonance image of rodent brain at high magnetic fields. Magn. Reson. Med..

[4] Mohajerani MH (2013). Spontaneous cortical activity alternates between motifs defined by regional axonal projections. Nat. Neurosci..

[5] Fiser J, Chiu C, Weliky M (2004). Small modulation of ongoing cortical dynamics by sensory input during natural vision. Nature.

[6] Chapman RM (2018). Temporospatial components of brain ERPs as biomarkers for Alzheimer’s disease. Alzheimer’s Dementia.

[7] Song M, Kang M, Lee H, Jeong Y, Paik SB (2018). Classification of spatiotemporal neural activity patterns in brain imaging data. Sci. Rep..

[8] Di Lorenzo F (2020). LTP-like cortical plasticity predicts conversion to dementia in patients with memory impairment. Brain Stimul..

[9] Barnikol UB (2006). Pattern reversal visual evoked responses of V1/V2 and V5/MT as revealed by MEG combined with probabilistic cytoarchitectonic maps. NeuroImage.

[10] Di Russo F, Martínez A, Sereno MI, Pitzalis S, Hillyard SA (2002). Cortical sources of the early components of the visual evoked potential. Human Brain Map..

[11] Orrison, W. & Lewine, J. *Functional Brain Imaging* Vol. 52 (Mosby, 1995).

[12] Babiloni C, Pizzella V, Gratta CD, Ferretti A, Romani GL (2009). Chapter 5 fundamentals of electroencefalography, magnetoencefalography, and functional magnetic resonance imaging. Int. Rev. Neurobiol..

[13] Mandal PK, Banerjee A, Tripathi M, Sharma A (2018). A comprehensive review of magnetoencephalography (MEG) studies for brain functionality in healthy aging and alzheimer’s disease (ad). Front. Comput. Neurosci..

[14] Olde Dubbelink KTE (2014). Disrupted brain network topology in Parkinson’s disease: A longitudinal magnetoencephalography study. Brain.

[15] Barik, K., Watanabe, K., Bhattacharya, J. & Saha, G. Classification of Autism in Young Children by Phase Angle Clustering in Magnetoencephalogram Signals. *2020 National Conference on Communications (NCC)* 1–6, 10.1109/NCC48643.2020.9056022 (2020)

[16] Dunkley BT, Jetly R, Pang EW, Taylor MJ (2020). New perspectives on the neurobiology of PTSD: High-resolution imaging of neural circuit (dys)function with magnetoencephalography. J. Milit. Veteran Family Health.

[17] Baillet S (2017). Magnetoencephalography for brain electrophysiology and imaging. Nat. Neurosci..

[18] Coquelet N (2020). Comparing MEG and high-density EEG for intrinsic functional connectivity mapping. NeuroImage.

[19] Gross J (2013). Comments and Controversies Good practice for conducting and reporting MEG research. NeuroImage.

[20] Allred JC, Lyman RN, Kornack TW, Romalis MV (2002). High-sensitivity atomic magnetometer unaffected by spin-exchange relaxation. Phys. Rev. Lett..

[21] Lin, C.-H. *et al.* Using optically-pumped magnetometers to measure magnetoencephalographic signals in the human cerebellum. *bioRxiv*10.1101/425447 (2018). 10.1113/JP277899 PMC6767854 31240719

[22] Boto E (2016). On the potential of a new generation of magnetometers for MEG: A beamformer simulation study. PLoS ONE.

[23] Tan S, Roth BJ, Wikswo JP (1990). The magnetic field of cortical current sources: The application of a spatial filtering model to the forward and inverse problems. Electroencephalogr. Clin. Neurophysiol..

[24] Boto E (2017). A new generation of magnetoencephalography: Room temperature measurements using optically-pumped magnetometers. NeuroImage.

[25] Iivanainen J, Stenroos M, Parkkonen L (2017). Measuring MEG closer to the brain: Performance of on-scalp sensor arrays. NeuroImage.

[26] Storm, J. H., Hömmen, P., Drung, D. & Körber, R. An ultra-sensitive and wideband magnetometer based on a superconducting quantum interference device. *Appl. Phys. Lett.* **110**, 10.1063/1.4976823 (2017).

[27] Kominis IK, Kornack TW, Allred JC, Romalis MV (2003). A subfemtotesla multichannel atomic magnetometer. Nature.

[28] Hochwald B, Nehorai A (1997). Magnetoencephalography with diversely oriented and multicomponent sensors. IEEE Trans. Biomed. Eng..

[29] Jbabdi S, Sotiropoulos SN, Savio AM, Graña M, Behrens TEJ (2012). Model-based analysis of multishell diffusion MR data for tractography: How to get over fitting problems. Magn. Reson. Med..

[30] Warrington S (2020). XTRACT: Standardised protocols for automated tractography in the human and macaque brain. NeuroImage.

[31] Sander, T., Jodko-Władzińska, A., Hartwig, S., Brühl, R. & Middelmann, T. *Optically pumped magnetometers enable a new level of biomagnetic measurements*10.1515/aot-2020-0027 (2020).

[32] Knappe, S., Sander, T. & Trahms, L. Optically-Pumped Magnetometers for MEG. In *Magnetoencephalography From Signals to Dynamic Cortical Networks*, vol. 9783642330, chap. Part VI, 993–999, 10.1007/978-3-642-33045-2 (Springer, 2014).

[33] Odom JV (2016). ISCEV standard for clinical visual evoked potentials: (2016 update). Documenta Ophthalmologica.

[34] Koessler L (2009). Automated cortical projection of EEG sensors: Anatomical correlation via the international 10–10 system. NeuroImage.

[35] Roland PE (2006). Cortical feedback depolarization waves: A mechanism of top-down influence on early visual areas. Proc. Natl. Acad. Sci. USA.

[36] Xu W, Huang X, Takagaki K, Wu J-Y (2007). Compression and reflection of visually evoked cortical waves. Neuron.

[37] Oostenveld R, Fries P, Maris E, Schoffelen J-M (2011). FieldTrip: Open source software for advanced analysis of meg, eeg, and invasive electrophysiological data. Comput. Intell. Neurosci..

[38] Foxe JJ (2008). Parvocellular and magnocellular contributions to the initial generators of the visual evoked potential: High-density electrical mapping of the C1 component. Brain Topogr..

[39] Pratt H, Bleich N, Martin WH (1995). Short latency visual evoked potentials to flashes from light-emitting diodes. Electroencephalogr. Clin. Neurophysiol. Evoked Potentials.

[40] Sharma R, Joshi S, Singh KD, Kumar A (2015). Visual evoked potentials: Normative values and gender differences. J. Clin. Diagn. Res..

[41] Jeffreys DA, Axford JG (1972). Source locations of pattern-specific components of human visual evoked potentials. I. Component of striate cortical origin. Exp. Brain Res..

[42] Coburn KL, Amoss RT, Arruda JE, Kizer LD, Marshall YS (2005). Effects of flash mode and intensity on P2 component latency and amplitude. Int. J. Psychophysiol..

[43] Brindley GS (1972). The variability of the human striate cortex. J. Physiol..

[44] Ahlfors SP (1999). Spatiotemporal activity of a cortical network for processing visual motion revealed by MEG and fMRI. J. Neurophysiol..

[45] Vanni S, Tanskanen T, Seppä M, Uutela K, Hari R (2001). Coinciding early activation of the human primary visual cortex and anteromedial cuneus. Proc. Natl. Acad. Sci..

[46] Yoshida F (2017). Noninvasive spatiotemporal imaging of neural transmission in the subcortical visual pathway. Sci. Rep..

[47] Babiloni C (2020). What electrophysiology tells us about Alzheimer’s disease: A window into the synchronization and connectivity of brain neurons. Neurobiol. Aging.

